# Accurate Needle Localization Using Two-Dimensional Power Doppler and B-Mode Ultrasound Image Analyses: A Feasibility Study

**DOI:** 10.3390/s18103475

**Published:** 2018-10-16

**Authors:** Mohammad I. Daoud, Ahmad Shtaiyat, Adnan R. Zayadeen, Rami Alazrai

**Affiliations:** 1 Department of Computer Engineering, German Jordanian University, Amman 11180, Jordan; Eng_stud3@yahoo.com (A.S.); rami.azrai@gju.edu.jo (R.A.); 2 Ultrasound Section, Jordanian Royal Medical Services, Amman 11180, Jordan; dr_adnan1978@yahoo.com

**Keywords:** ultrasound-guided needle interventions, needle localization, B-mode ultrasound imaging, power Doppler ultrasound imaging, medical ultrasound image and signal processing

## Abstract

Curvilinear ultrasound transducers are commonly used in various needle insertion interventions, but localizing the needle in curvilinear ultrasound images is usually challenging. In this paper, a new method is proposed to localize the needle in curvilinear ultrasound images by exciting the needle using a piezoelectric buzzer and imaging the excited needle using a curvilinear ultrasound transducer to acquire a power Doppler image and a B-mode image. The needle-induced Doppler responses that appear in the power Doppler image are analyzed to estimate the needle axis initially and identify the candidate regions that are expected to include the needle. The candidate needle regions in the B-mode image are analyzed to improve the localization of the needle axis. The needle tip is determined by analyzing the intensity variations of the power Doppler and B-mode images around the needle axis. The proposed method is employed to localize different needles that are inserted in three *ex vivo* animal tissue types at various insertion angles, and the results demonstrate the capability of the method to achieve automatic, reliable and accurate needle localization. Furthermore, the proposed method outperformed two existing needle localization methods.

## 1. Introduction

Ultrasound is among the most widely-used medical imaging modalities for guiding minimally-invasive needle insertion interventions [1,2,3]. In fact, ultrasound guidance of the needle provides the ability to observe the advancement of the needle towards the target anatomy in real time, which can improve the accuracy, increase the success rate, and reduce the cost of the intervention [4]. The use of ultrasound imaging to guide needle insertion interventions is attributed to several advantages of ultrasound, including the low cost, safety, real-time imaging capability and portability [5]. However, visualizing the needle in conventional brightness mode (B-mode) ultrasound images might be challenging due to various factors. Examples of these factors include the ultrasound speckle interference pattern that affects the quality of ultrasound images and the presence of strong linear anatomical structures in the ultrasound image, such as bone, that have characteristics similar to the needle [6,7]. Furthermore, steep needle insertion angles, which are common in many medical procedures, might reduce the visibility of the needle in B-mode ultrasound images due to needle reflection of the ultrasound beam away from the transducer [7,8].

Many methods have been proposed to localize the needle using B-mode ultrasound image analysis. An example of these methods is the algorithm introduced by Ding and Fenster [9] that used a fast implementation of the Hough transform based on a coarse-fine search approach to localize the needle in B-mode ultrasound images. Kaya et al. [10] employed Gabor filtering to localize the needle in B-mode ultrasound images. In particular, the B-mode image is processed using a Gabor filter [11], dynamic thresholding and the robust model fitting random sample consensus (RANSAC) algorithm to localized the needle axis. The tip of the needle is determined by defining a region of interest (ROI) in the filtered, binarized image and determining the strongest edge in the deepest blob that is located at the needle axis. Hacihaliloglu et al. [12] investigated the problem of localizing the needle in B-mode images acquired using a two-dimensional (2D) curvilinear ultrasound transducer. In fact, the visibility of the needle in curvilinear B-mode images is mainly limited to a portion of the needle that is orthogonal or nearly orthogonal to the ultrasound beams [12,13]. The method by Hacihaliloglu et al. [12] aimed to localize the needle axis and tip in curvilinear B-mode images by extending the visible part of the needle, which is assumed to be located at a specific region in the ultrasound image. In particular, the region that includes the visible part of the needle is processed using projection-based local phase analysis and Radon transform to obtain an initial estimation of the needle axis and identify an approximate region that surrounds the needle axis. This approximate region is processed using local phase analysis and the maximum likelihood estimation sample consensus (MLESAC) algorithm to improve the accuracy of estimating the needle axis. Finally, the position of the needle tip along the estimated needle axis is determined using image statistical analysis. Mwikirize et al. [14] introduced a method for enhancing the needle visibility and localizing the needle in curvilinear B-mode images. In this method, the visibility of the needle is enhanced by computing signal attenuation maps that model ultrasound attenuation and scattering. Moreover, a fixed region is defined in the enhanced ultrasound image, such that this region is located at the insertion site of the needle and includes part of the needle shaft. The needle axis is estimated based on this region using local phase analysis, Radon transform and the MLESAC algorithm. Moreover, the needle tip is estimated using statistical analysis that is similar to the analysis employed in [12]. In fact, the assumptions employed by the needle localization methods in [12,14], which include the assumptions about the a priori known insertion location of the needle and the assumption about the location of the visible part of the needle, might limit their application in real-life clinical procedures. In general, the performance of the needle localization methods that are based on B-mode image analysis is affected by the visibility of the needle in the B-mode ultrasound image. Hence, many of the B-mode needle localization methods achieve good results when the needle is the predominant linear reflector in the ultrasound image.

Curvilinear ultrasound transducers are commonly used in many needle insertion interventions due to their capability to obtain wide fields of view and enable large penetration depths [15,16]. Hence, accurate and reliable localization of the needle in curvilinear ultrasound images is an important and challenging research problem. To address this research problem, Daoud et al. [13,17] proposed a novel method for localizing the needle in curvilinear ultrasound images. In this method, the elements of the curvilinear ultrasound transducer are employed to generate a circular ultrasound wave, and the pre-beamformed ultrasound radio-frequency (RF) signal received by each individual transducer element is recorded. The pre-beamformed RF signals are analyzed to localize the echoes received from the needle using signature-based, needle-specific features. The axis of the needle in the curvilinear ultrasound image is estimated by fitting the arrival times of the echoes received from the needle to a model that describes needle reflection of circular ultrasound waves. Despite the high accuracy and reliability of estimating the needle axis that is achieved using this method, the current formulation of the method does not support the localization of the needle tip. Moreover, the recording of the pre-beamformed ultrasound RF signals requires dedicated parallel acquisition hardware that might not be available in many commercial ultrasound imaging systems.

An attractive approach to enhance the needle visibility and improve the localization of the needle in ultrasound images is to excite the needle mechanically and employ Doppler ultrasound imaging to detect the Doppler responses induced by the vibrating needle [18]. This Doppler-based approach has been employed in several studies, such as [18,19,20], to localize the needle with accuracies on the order of a few millimeters. For example, Adebar et al. [20] showed that the needle visibility obtained by employing power Doppler ultrasound to image a needle excited using low-amplitude, high-frequency acoustic vibrations, which are generated by a small piezoelectric buzzer mounted near the needle base, is higher than the needle visibility achieved by conventional B-mode ultrasound images. Despite the improved needle visibility obtained using this Doppler-based approach, the irregular Doppler responses that are induced around the excited needle can mainly be employed to achieve approximate localization of the needle [21]. This limitation can be addressed by combining Doppler ultrasound imaging of the excited needle, which enables improved needle visibility, but with limited localization accuracy, and B-mode ultrasound imaging, which supports accurate detection of the needle, but might have limited needle visibility, to achieve effective needle localization. Such a hybrid approach has been employed by Greer et al. [21], who combined power Doppler and B-mode ultrasound image analyses to obtain three-dimensional (3D) segmentation of a curved needle using a tracked 2D ultrasound transducer. In particular, the method presented in [21] is composed of two phases: image analysis and curve fitting. In the image analysis phase, the 2D power Doppler and B-mode ultrasound images are analyzed to obtain candidate needle points. The image analysis phase starts by processing the power Doppler ultrasound image to identify ROIs in the image, such that each ROI has accumulative intensity greater than an experimentally-defined threshold. Moreover, the ROIs are clustered, such that each group of neighboring ROIs is combined into one cluster. The clustered ROIs define potential regions in the B-mode image that are analyzed using dynamic thresholding and eigenvector analysis to localize the candidate needle points. The candidate needle points are mapped to the 3D spatial coordinate system based on the tracked position of the ultrasound transducer. In the curve fitting phase, curve fitting analysis is used to determine the 3D location of the needle based on the candidate needle points. In fact, the method presented in [21] can be readily customized to enable needle localization in 2D ultrasound images by eliminating the mapping of the candidate needle points, which are initially localized in the 2D ultrasound image plane, to the 3D spatial coordinate system. However, the method by Greer et al. [21] did not investigate the use of the combined power Doppler and B-mode ultrasound image analyses to accurately localize straight needles at different needle insertion angles.

In this study, a new method is proposed to automatically localize straight needles, which are denoted here for simplicity as needles, in 2D curvilinear ultrasound images with high accuracy and reliability. In fact, the proposed method combines both power Doppler image analysis and B-mode image analysis. The experimental setup employed to localize the needle is close to the study by Greer et al. [21], in which the needle is excited using a piezoelectric buzzer mounted near the needle base. The excited needle is imaged using a 2D curvilinear ultrasound transducer to acquire a power Doppler ultrasound image and a B-mode ultrasound image. The acquired power Doppler ultrasound image, which aims to detect the needle-induced Doppler responses, is analyzed to obtain an initial estimation of the needle axis and determine candidate regions in the ultrasound image that are expected to include the needle. The accuracy of estimating the needle axis is improved by analyzing the B-mode ultrasound image, such that the B-mode image analysis is restricted to the candidate regions that are expected to include the needle. The location of the needle tip is determined by analyzing the intensity variations of the power Doppler and B-mode ultrasound images around the accurately-localized needle axis. The feasibility of the proposed method is demonstrated by localizing the axes and tips of needles inserted in three different *ex vivo* animal tissue types at shallow, moderate and steep needle insertion angles and comparing the results with matching ground truth needle segmentations. Furthermore, the performance of our proposed method is compared with the needle localization methods introduced by Hacihaliloglu et al. [12] and Greer et al. [21], where the former provides a well-studied method for localizing the needle using B-mode ultrasound image analysis, and the latter, to the best of our knowledge, is the only computer-based needle localization method reported in the literature that combines power Doppler and B-mode ultrasound image analyses. In fact, the main contribution of the current study is the improved power Doppler and B-mode ultrasound image analyses that enable automatic, reliable and accurate localization of the needle axis and tip in 2D curvilinear ultrasound images for an extended range of needle insertion angles. Moreover, the proposed method achieves effective differentiation of the needle from other linear, needle-like reflectors that might appear in the ultrasound image.

The rest of the paper is organized as follows. Section 2 provides a detailed description of the proposed needle localization method. Section 3 presents the experimental setup and evaluation procedures that are employed to assess the accuracy of the proposed method and compare its performance with the two previous needle localization methods in [12,21]. The experimental results are provided in Section 4. Finally, the discussion and conclusion are provided in Section 5 and Section 6, respectively.

## 2. Methods

### 2.1. Overview of the Proposed Needle Localization Method

**Figure 1:**
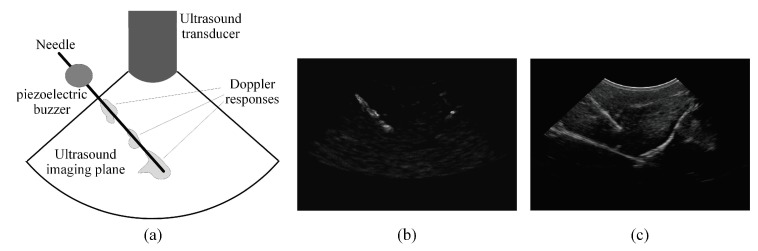
(**a**) Schematic representation of the needle imaging setup. In this setup, a piezoelectric buzzer is mounted near the needle base to generate acoustic waves that vibrate the needle. (**b**,**c**) An ultrasound imaging system equipped with a curvilinear transducer is employed to acquire (**b**) a power Doppler image and (**c**) a B-mode image of the excited needle.

Similar to the experimental procedure employed in [21], a small piezoelectric buzzer is mounted near the base of the needle as illustrated in Figure 1a. The buzzer aims to generate low-amplitude, high-frequency acoustic waves that excite the needle. The excited needle is scanned using a curvilinear ultrasound transducer to acquire a power Doppler image and a B-mode image. For illustration, Figure 1b,c presents a power Doppler image and a B-mode image, respectively, that are acquired for an excited needle inserted in an *ex vivo* bovine muscle specimen. As shown in Figure 1b, the irregular Doppler responses generated by the excited needle are detected by the power Doppler image. These irregular power Doppler responses are analyzed to obtain an approximate, or initial, estimation of the needle axis and identify the candidate regions that are expected to include the needle. The B-mode ultrasound image is analyzed to achieve accurate localization of the needle axis, such that the B-mode image analysis is guided based on the initially estimated needle axis and the candidate regions of the needle that are obtained using the power Doppler image analysis. The tip of the needle is determined based on both the power Doppler image and the B-mode image by analyzing the intensity variations in these two ultrasound images around the accurately localized needle axis.

### 2.2. Analyzing the Power Doppler Ultrasound Image to Obtain an Initial Estimation of the Needle Axis and Identify the Candidate Regions of the Needle

To obtain an initial estimation of the needle axis, the power Doppler ultrasound image is preprocessed using a thresholding approach to eliminate all pixels with small intensity values since they indicate weak Doppler responses. To achieve this goal, a threshold value is computed by applying the iterative intermeans dynamic thresholding method [22] on the power Doppler image. All pixels in the power Doppler image that are smaller than the threshold are set to zero, and the remaining pixels are unaltered.

After preprocessing, the nonzero pixels of the power Doppler ultrasound image are grouped into clusters. The clustering is performed using the density-based spatial clustering of applications with noise (DBSCAN) [23] algorithm. The DBSCAN algorithm has two control parameters, which are the maximum distance between the core of the cluster and any pixel inside the cluster, denoted by *R*, and the minimum number of pixels in each individual cluster, denoted by *M*. The tuning of these two control parameters is described in Section 3.2. The non-clustered pixels, i.e., the pixels that do not satisfy these two control parameters and hence do not belong to any cluster, are considered as noise [23]. The clustering process aims to group the pixels of the power Doppler image into clusters that represent regions with active Doppler responses. Hence, these clusters represent potential regions that are expected to include the exited needle.

An initial estimation of the needle axis is obtained by identifying the longest and strongest line that passes through the clusters of the power Doppler image. In this study, the detection of the strongest and longest line is achieved by applying the Radon transform [24] to the pixels that belong to the clusters in the power Doppler image using integration angles between $0^{\circ }$ and $359^{\circ }$, with an increment of $1^{\circ }$. The maximum value of the Radon transform computed over this range of angles corresponds to the strongest and longest line in the image. Hence, this maximum Radon transform value is extracted and projected back using the inverse Radon transform to obtain a line in the image that represents the initially estimated needle axis.

**Figure 2:**
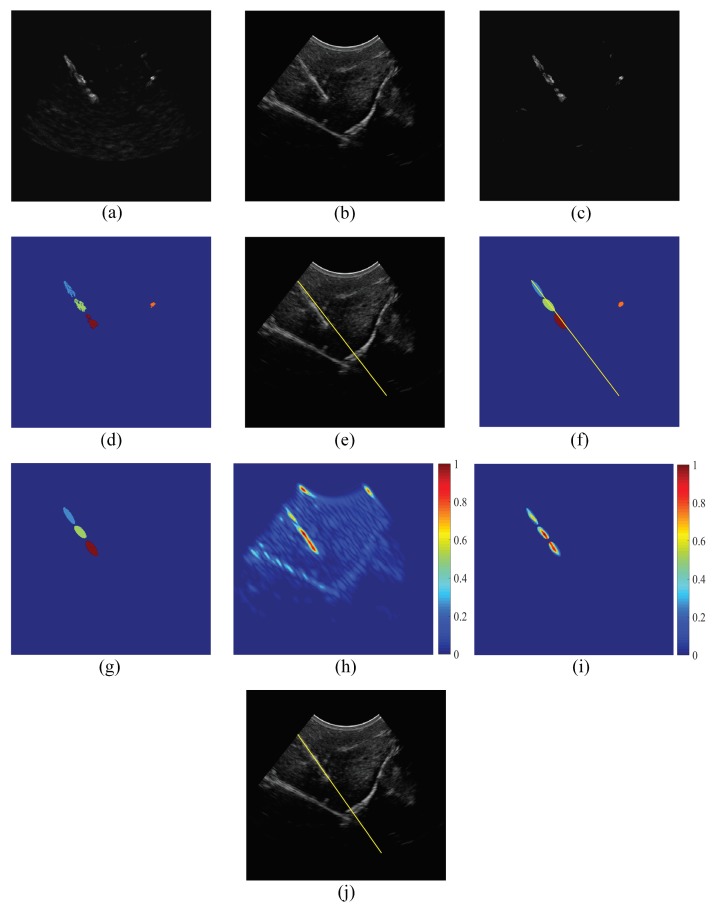
An illustrative example that shows the stages employed by the proposed method to localize the needle axis.

For example, consider the power Doppler image and the B-mode image in Figure 2a,b, respectively, that are acquired for an excited needle inserted in an *ex vivo* bovine muscle specimen. The Doppler-based analyses that are performed to obtain an initial estimation of the needle axis are illustrated in Figure 2c–e. In particular, Figure 2c shows the image obtained after preprocessing the power Doppler image in Figure 2a to eliminate the weak Doppler responses using the iterative intermeans dynamic thresholding method. Figure 2d presents the clusters obtained by decomposing the preprocessed image in Figure 2c using the DBSCAN algorithm. The initially estimated needle axis, which is computed based on the clusters in Figure 2d, is shown in Figure 2e as a line overlaid on the B-mode ultrasound image. As shown in Figure 2e, the initially estimated needle axis is close to the axis of the true needle.

As described previously, the clusters obtained using the power Doppler image analysis correspond to candidate regions that are expected to include the needle. Hence, the accuracy of needle localization can be improved by searching for the needle within the regions that correspond to these clusters. However, employing the clusters of the power Doppler image, which have irregular shapes, during the analysis to improve needle localization might impose high computational complexity. To address this limitation, the irregular clusters of the power Doppler image are represented using regular-shaped ellipses. The representation of a given cluster is performed by computing three geometric parameters, which are the center point, major axis and minor axis of the cluster. Each cluster is represented by an ellipse that has the same values of the three geometric parameters. Since the initially estimated needle axis is expected to be close to the axis of the true needle, the ellipses located away from the initially estimated needle axis are considered false positive regions that should not be included in the needle localization analysis. In particular, all ellipses that do not intercept with the initially estimated needle axis are eliminated. Figure 2f shows the ellipses that represent the irregular clusters in Figure 2d as well as the initially estimated needle axis. The remaining ellipses after applying the ellipse elimination procedure described above are presented in Figure 2g. In fact, the remaining ellipses provide regions of interest (ROIs) that are expected to include the true needle. Hence, these remaining ellipses, which are called the power Doppler-based ROIs, are employed to refine the localization of needle axis, as described in the next subsection.

### 2.3. Analyzing the B-Mode Ultrasound Image to Obtain Accurate Localization of the Needle Axis

The ultrasound B-mode image is analyzed to obtain accurate localization of the needle axis. In fact, the B-mode image analysis is guided based on the assumption that the true needle is located close to the initially estimated needle axis and passes through all or a subset of the power Doppler-based ROIs. In particular, the B-mode ultrasound image is processed to enhance the visibility of all linear structures that have a scale close to the needle size and orientation close to the initially estimated needle axis. This process of enhancing the B-mode image is performed by filtering the image using a Gabor filter [11]. The Gabor filter provides an orientation-specific line detector that maximizes the intensity of the linear structures in the image that match its scale and orientation [25]. In the spatial domain, the 2D Gabor filter can be defined as a multiplication of a 2D Gaussian envelope and a complex sinusoidal plane wave [26]:

(1) $$g\left(x,y\right)=e^{-\frac{1}{2}\left(\frac{{x^{\prime }}^{2}}{\sigma_{x}^{2}}+\frac{{y^{\prime }}^{2}}{\sigma_{y}^{2}}\right)}e^{\frac{j2\pi x^{\prime }}{\lambda }}$$

where $x^{\prime }=x\;\cos\left(\theta \right)+y\;\sin\left(\theta \right)$, $y^{\prime }=-x\;\sin\left(\theta \right)+y\;\cos\left(\theta \right)$, θ is the orientation of the Gabor filter, $\sigma_{x}$ and $\sigma_{y}$ are the standard deviations of the Gaussian envelope along the $x^{\prime }$ and $y^{\prime }$ dimensions and λ is the wavelength of the complex exponential signal $e^{j2\pi x^{\prime }/\lambda }=\cos\left(j2\pi x^{\prime }/\lambda \right)+j\sin\left(j2\pi x^{\prime }/\lambda \right)$. The orientation of the Gabor filter, θ, is configured to match the orientation of the initially estimated needle axis, which is obtained using the power Doppler image analysis. The wavelength, λ, determines the width of the line detector achieved by the Gabor filter [27]. Hence, the value of λ is set to match the needle diameter. The standard deviation along the $x^{\prime }$ dimension is set to $\sigma_{x}=\lambda /\left(2\sqrt{2ln2}\right)$, such that the amplitude of the 2D Gaussian envelop in Equation (1) is reduced to 50% of its maximum amplitude at $x^{\prime }=\lambda /2$ and $y^{\prime }=0$ [27]. Moreover, the standard deviation along the $y^{\prime }$ dimension is set to $\sigma_{x}=\lambda /\left(\sqrt{2ln2}\right)$, such that the amplitude of the Gaussian envelop is reduced to 50% of its maximum amplitude at $x^{\prime }=0$ and $y^{\prime }=\lambda$ [27]. These parameters of the Gabor filter aim to enhance the visibility of the structures in the B-mode image that match the scale and orientation of the needle. For example, Figure 2h shows the image obtained after filtering the B-mode image in Figure 2b using the Gabor filter. As shown in Figure 2h, the intensities of the linear and semi-linear structures in the B-mode image that are close to the scale and orientation of the needle are improved after applying the Gabor filter. However, analyzing the filtered image to achieve accurate localization of the needle axis might be challenging since the image can include various linear and semi-linear structures with scales and orientations that are close to the needle. To overcome this potential limitation, the filtering of the B-mode image using the Gabor filter is masked based on the power Doppler-based ROIs. In particular, the masking is performed by applying the Gabor filter to the regions in the B-mode image that correspond to these ROIs and assigning a value of zero to the regions in the B-mode image that are located outside the ROIs. Figure 2i shows the outcome of applying the Gabor filter to the B-mode image in Figure 2b, such that the filtering process is masked using the power Doppler-based ROIs in Figure 2g.

The filtered B-mode image is analyzed to localize the needle axis accurately. In particular, the Radon transform is computed for the filtered B-mode image using the angle of the initially estimated needle axis, which is obtained based on the power Doppler image analysis. The maximum value of the Radon transform at this angle is determined and projected back to the spatial domain using the inverse Radon transform to obtain a line in the ultrasound image coordinate system that represents an accurate estimation of the needle axis. For example, the accurately estimated needle axis, which is obtained based on the filtered B-mode image in Figure 2i, is shown in Figure 2j as a yellow line overlaid on the B-mode ultrasound image.

### 2.4. Analyzing the Power Doppler and B-Mode Ultrasound Images to Obtain Accurate Localization of the Needle Tip

**Figure 3:**
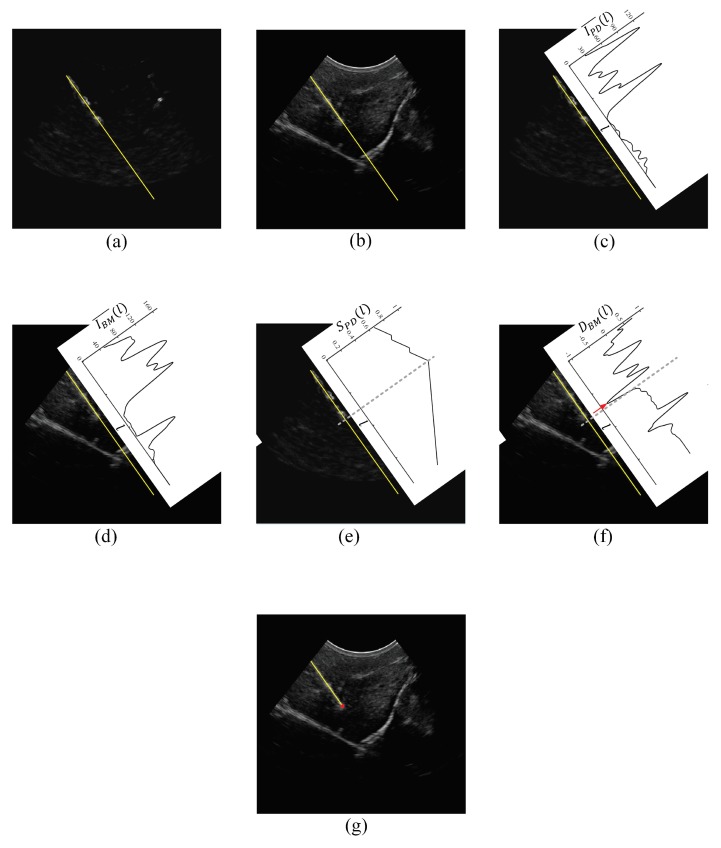
An illustrative example that shows the stages employed by the proposed method to localize the needle tip.

The location of the needle tip is determined by employing a moving window approach to analyze the intensities of the power Doppler image and the B-mode image around the accurately localized needle axis. The window has a rectangular shape, a length that matches two-times the needle diameter, and a width that is equal to the needle diameter. The window is initially positioned such that its center is located at the entry point of the needle and its long side is parallel to the needle axis. The average values of the power Doppler intensity and the B-mode intensity within the window are computed and assigned to the pixel located at the center of the window. In this study, the average value of the power Doppler intensity and the average value of the B-mode intensity within the window are denoted by $\bar{I_{PD}}\left(l\right)$ and $\bar{I_{BM}}\left(l\right)$, respectively, where *l* represents the center pixel of the window that is located at the needle axis. The location of the window is shifted by one pixel along the needle axis, and the process of computing the average values of the power Doppler intensity, $\bar{I_{PD}}\left(l\right)$ and B-mode intensity, $\bar{I_{BM}}\left(l\right)$, within the window and assigning these two values to the pixel, *l*, at the window’s center is repeated until the entire needle axis is traversed. For example, Figure 3a,b shows the power Doppler image and the B-mode image presented in Figure 2a,b, respectively, along with the accurately localized needle axis (yellow line). The functions $\bar{I_{PD}}\left(l\right)$ and $\bar{I_{BM}}\left(l\right)$ that are computed for the power Doppler image and the B-mode image are shown in Figure 3c,d, respectively.

The power Doppler responses induced by the needle, and particularly the excited needle tip, can be used to achieve robust identification of the needle tip, as suggested in [18]. However, the irregular nature of the needle-induced power Doppler responses limits the localization accuracy of the needle tip. In this study, an approximate localization of the needle tip is obtained by traversing the needle axis starting from the entry point of the needle and analyzing the needle-induced power Doppler responses. During the transverse process, the needle tip is considered as a termination point for the needle-induced power Doppler responses. In fact, the approximate identification of the needle tip based on the needle-induced power Doppler responses is carried out using a two-phase approach. In the first phase, a thresholding procedure is employed to identify the needle-induced power Doppler responses, which are expected to have relatively large amplitudes compared to the other responses that exist in the power Doppler image. In this procedure, a threshold, denoted by Th, is computed as the mean value of $\bar{I_{PD}}\left(l\right)$. The values of $\bar{I_{PD}}\left(l\right)$ that are greater than or equal to Th are considered to correspond to the strong needle-induced power Doppler responses. Moreover, the values of $\bar{I_{PD}}\left(l\right)$ that are smaller than Th are not expected to be related to the strong needle-induced power Doppler responses. Hence, a binary function, denoted by $B_{PD}\left(l\right)$, is defied to identify the location of the strong needle-induced power Doppler responses, as follows:

(2) $$B_{PD}\left(l\right)=\left\{\begin{array}{c} 1,\;\;\;\bar{I_{PD}}\left(l\right)\geq Th\\ 0,\;\;\;\bar{I_{PD}}\left(l\right)<Th \end{array}\right.$$

A value of $B_{PD}\left(l\right)$ that is equal to one indicates strong needle-induced power Doppler responses at *l*, and a value of $B_{PD}\left(l\right)$ that is equal to zero indicates weak power Doppler responses at *l* that might not be related to the needle. The second phase of the approximate needle tip localization approach aims to analyze $B_{PD}\left(l\right)$ with the goal of finding the tip of the needle that separates the strong needle-induced power Doppler responses from the weak power Doppler responses that are not related to the needle. To localize the needle tip, a function, denoted by $S_{PD}\left(l\right)$, is defined to compute the fraction of the pixels along the needle axis that are located before *l* and have strong power Doppler responses plus the fraction of the pixels along the needle axis that are located after *l* and have weak power Doppler responses, as follows:

(3) $$S_{PD}\left(l\right)=\frac{\sum_{i=0}^{l}B_{PD}\left(i\right)+\sum_{i=l+1}^{L}\left(1-B_{PD}\left(i\right)\right)}{L}$$

where *L* is the total length of the localized needle axis expressed in pixels. An approximate localization of the needle tip is achieved by finding the peak in $S_{PD}\left(l\right)$ that has the highest value. In fact, this peak corresponds to a termination point that separates the strong needle-induced power Doppler responses from the weak power Doppler responses that are not related to the needle. For example, Figure 3e presents the function $S_{PD}\left(l\right)$ that is computed for the power Doppler image in Figure 3a. As shown in Figure 3e, the peak of $S_{PD}\left(l\right)$ that has the highest value, which is indicated by the dashed gray line, is located close to the needle tip. As described previously, the irregular nature of the power Doppler responses generated by the excited needle limits the tip localization accuracy that can be achieved using $S_{PD}\left(l\right)$.

The accuracy of localizing the needle tip is improved by analyzing the B-mode image. In fact, the needle tip in the B-mode image represents a disconnection point along the needle axis, such that this disconnection point separates the bright pixels of the needle from the pixels of the background tissue. Hence, the derivative of $\bar{I_{BM}}\left(l\right)$, denoted by $D_{BM}\left(l\right)=\left|\frac{d\bar{I_{BM}}\left(l\right)}{dl}\right|$, is computed to quantify the discontinuities in the B-mode image pixel intensities along the needle axis. The needle tip corresponds to a strong negative peak in $D_{BM}\left(l\right)$ that is located close to the approximate location of the needle tip obtained using the power Doppler image analysis. In this study, accurate localization of the needle tip is achieved by identifying the strongest negative peak in $D_{BM}\left(l\right)$ that is separated by a distance smaller than two-times the needle diameter from the approximate location of the needle tip obtained using the power Doppler image analysis. For example, Figure 3f shows the function $D_{BM}\left(l\right)$ that is computed for the B-mode image in Figure 3b. In this figure, the approximate location of the needle tip obtained using the power Doppler image analysis is shown as a dashed gray line. Accurate localization of the needle tip is obtained by finding the strongest negative peak in $D_{BM}\left(l\right)$ that is located close to the dashed gray line, where the red arrow in Figure 3f points to this negative peak. The localized needle tip is shown in Figure 3g as a red circle overlaid on the B-mode ultrasound image.

## 3. Experiments

### 3.1. Experimental Setup

**Figure 4:**
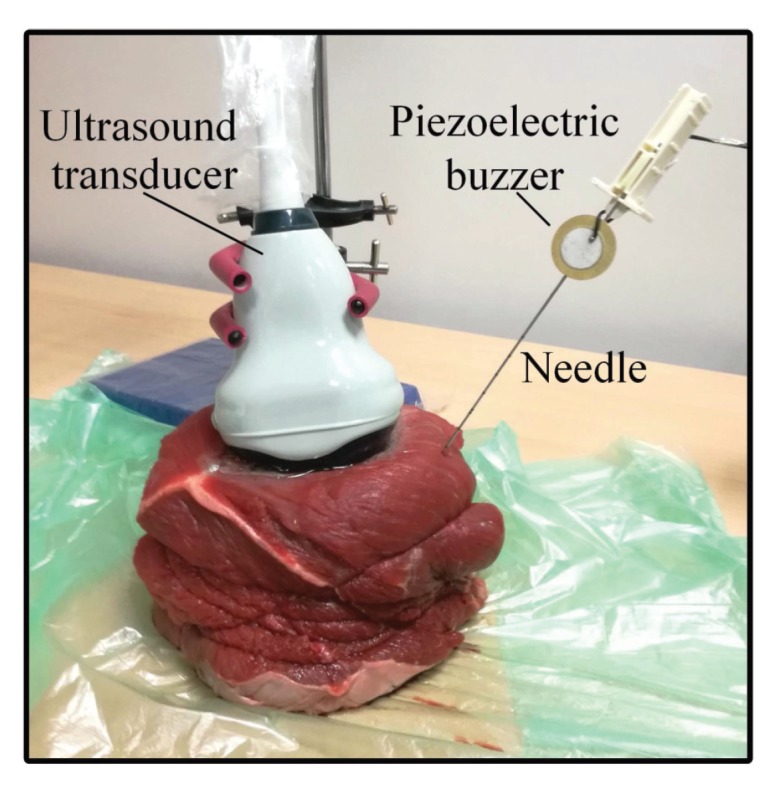
The experimental setup.

A set of *ex vivo* ultrasound needle imaging experiments was performed to evaluate the performance of the proposed needle localization method. The setup employed in these experiments is shown in Figure 4. In this setup, a needle is inserted in a freshly-excised *ex vivo* animal tissue specimen, such that the inserted needle is excited using a piezoelectric buzzer (AB2720B, PUI Audio Inc., Dayton, OH, USA) mounted near its base as described in Section 2.1. The buzzer was derived at its resonance frequency, which is equal to 2 kHz, using a square wave generated by a function generator (Model 4017A, B&K Precision Inc., Yorba Linda, CA, USA). A SonixTouch Q+ ultrasound imaging system (BK Ultrasound, Peabody, MA, USA) equipped with a 4DC7-3/40 curvilinear transducer was used to acquire a pair of 2D power Doppler and 2D B-mode ultrasound images for the inserted needle. The frequency of the B-mode ultrasound imaging is set to 6.5 MHz, and the frequency of the power Doppler ultrasound imaging is set to 3.5 MHz. Moreover, the imaging depth of the ultrasound system is set to 90 mm. During each imaging trial, the ultrasound transducer is fixed using a mounting tool, and the needle is held by hand. The needles used in the experiments are standard 18G and 16G biopsy needles with diameters of 1.27 mm and 1.65 mm, respectively. Furthermore, the *ex vivo* animal tissue types employed in the experiences are bovine muscle, bovine liver and porcine muscle, where these tissue types have different echogenicity characteristics.

As suggested in [20,28], power Doppler ultrasound imaging is performed with the wall motion filter set to its maximum value to reduce the low-frequency Doppler signals produced by unwanted tissue motion with respect to the ultrasound transducer and maximize the capability of detecting the high-frequency Doppler signals generated by the vibrating needle. The pulse repetition frequency (PRF) is tuned manually by setting its value to 0.2, 0.3, 0.4, 0.5, 0.6, 0.7, 1.0, 1.3, 1.4, 1.7, 2.0, 2.5, 3.3, 4.0, 5.0, 6.7 and 9.0 kHz, where these values are the PRF configurations provided by the manufacturing company. For each examined PRF value, the ultrasound system is employed to acquire three power Doppler images when an 18G needle is inserted in each one of the three tissue type (i.e., nine power Doppler images are acquired for the three tissue types). Since the visibility of the needle in the ultrasound image is affected by the needle insertion angle, we have varied the insertion angle of the needle during the acquisition of the power Doppler images. In particular, the three power Doppler images collected for each tissue type are configured such that one image is acquired when the needle is inserted at a shallow angle ($0^{\circ }$–$20^{\circ }$) with respect to the skin, one image is acquired at a moderate needle insertion angle ($20^{\circ }$–$40^{\circ }$) and one image is acquired at a steep needle insertion angle ($40^{\circ }$–$65^{\circ }$). The PRF value that enabled the highest needle visibility in the power Doppler images acquired for the three tissue types is equal to 1.7 kHz. In fact, when the PRF is equal to 1.7 kHz, the maximum value of the wall motion filter is 250 Hz. Hence, the PRF and wall motion filter values of 1.7 kHz and 250 Hz, respectively, are employed to perform the needle imaging experiments described in the following subsections.

### 3.2. Tuning the Parameters of the DBSCAN Clustering Algorithm

The power Doppler ultrasound image analysis, which aims to obtain an initial estimation of the needle axis as described in Section 2.2, is affected by the parameters *R* and *M* of the DBSCAN clustering algorithm. Hence, these parameters of the DBSCAN clustering algorithm are tuned to enable effective localization of the needle axis. The tuning process is performed using nine pairs of power Doppler and B-mode ultrasound images, such that three image pairs are collected when an 18G needle is inserted in each one of the three animal tissue types. Moreover, the three pairs of power Doppler and B-mode ultrasound images obtained for each tissue type are configured such that one image pair is acquired at a shallow needle insertion angle, one image pair is collected at a moderate needle insertion angle and one image pair is collected at a steep needle insertion angle. To obtain the ground truth segmentation of the needle in a given pair of power Doppler and B-mode ultrasound images, a sonographer (third author) with more than eleven years of experience was asked to segment the needle in the B-mode image five times. The ground truth segmentation of the needle is taken as the average of the five manual segmentations. Moreover, a validation procedure is applied to confirm the ground truth segmentation of the needle. This validation procedure is carried out after finishing the excitation of the needle and the acquisition of the pair of power Doppler and B-mode ultrasound images. In this procedure, the needle is rotated around its long axis to improve the visibility of the tip, as suggested in [29], and the ultrasound imaging system is employed to acquire a B-mode ultrasound video for the rotating needle. The location of the needle tip in the video is determined and compared with the needle tip in the ground truth needle segmentation.

**Figure 5:**
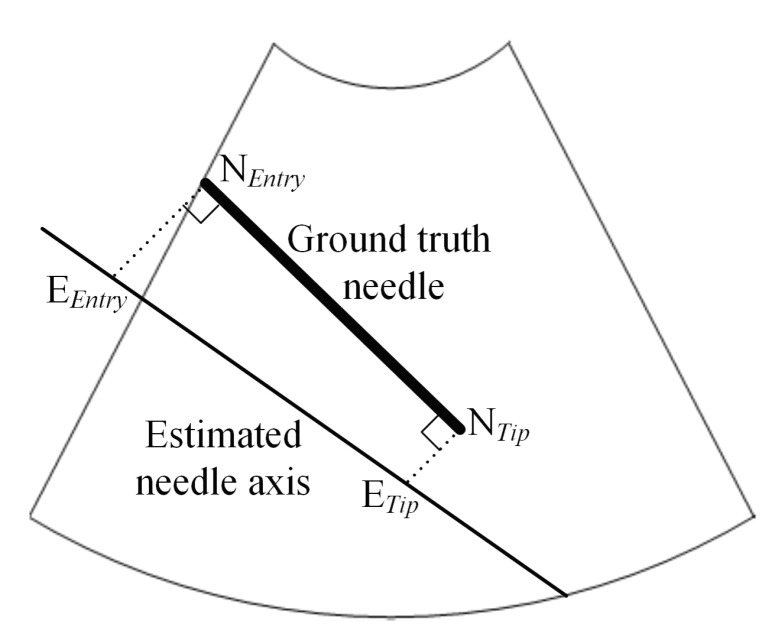
Graphical illustration of the computation of the axis error.

The tuning of the DBSCAN clustering algorithm is carried out using a grid search approach, in which *R* is varied between 1 and 10, incrementing by 1, and *M* is varied between 25 and 200, with an increment of 25. The grid search is configured to determine the values of *R* and *M* that minimize the mean axis error value of the initially estimated needle axes that are computed for the nine power Doppler ultrasound images, as described in Section 2.2. In fact, the calculation of the needle axis error is illustrated in Figure 5. In this figure, the points N${}_{Entry}$ and N${}_{Tip}$ are the entry point and the tip, respectively, of the ground truth needle. The orthogonal projections of N${}_{Entry}$ and N${}_{Tip}$ on the estimated needle axis are denoted by E${}_{Entry}$ and E${}_{Tip}$, respectively. The Euclidean distance between N${}_{Entry}$ and E${}_{Entry}$ is denoted by $∥N_{Entry},E_{Entry}∥$, and the Euclidean distance between N${}_{Tip}$ and E${}_{Tip}$ is denoted by $∥N_{Tip},E_{Tip}∥$. The axis error of the initially estimated needle axis is equal to the maximum of $∥N_{Entry},E_{Entry}∥$ and $∥N_{Tip},E_{Tip}∥$. The tuned values of the parameters *R* and *M* obtained using the grid search approach are equal to 6 and 100, respectively. Hence, these parameter values of the DBSCAN clustering algorithm are employed to perform the power Doppler ultrasound image analysis.

### 3.3. Performance Evaluations and Comparisons

To evaluate the performance of the proposed needle localization method, a set of 117 pairs of power Doppler and B-mode ultrasound images are acquired for 18G needles inserted in the three *ex vivo* animal tissue types. In fact, among these 117 image pairs, 39 image pairs are acquired for needles inserted in bovine muscle tissue, 39 image pairs are acquired for needles inserted in bovine liver tissue and 39 image pairs are acquired for needles inserted in porcine muscle tissue. During the acquisition of the ultrasound image pairs, the values of the needle insertion angle with respect to the skin were uniformly distributed between $0^{\circ }$ and $65^{\circ }$. Hence, for each one of the three tissue types, 12 pairs of power Doppler and B-mode ultrasound images are acquired at shallow needle insertion angles, 12 image pairs are acquired at moderate needle insertion angles and 15 image pairs are acquired at steep needle insertion angles. Moreover, the insertion depths of the needles are approximately uniformly distributed between 40 and 80 mm. In particular, for each tissue type, between 8 and 11 image pairs are acquired at each one of the following ranges of the needle insertion depths: 40–50 mm, 50–60 mm, 60–70 mm and 70–80 mm.

The acquired pairs of power Doppler and B-mode ultrasound images are analyzed using our proposed method to localize the needle in each image pair. Moreover, the ground truth needle in each image pair is determined using manual segmentation, as described in Section 3.2. The needle estimations obtained using our proposed method are compared with the matching ground truth needle segmentations. The comparison is performed using four error metrics: the failure rate, the needle axis error, the needle angle error and the needle tip error. The failure rate depends on the needle axis error, where the axis error is calculated as described in Section 3.2. In fact, the failure rate is defined as the percentage of needle localization trials in which the proposed method fails to localize the needle with an axis error value smaller than 3 mm. The computation of the angle error is performed by calculating the difference between the angle of the needle that is estimated using our proposed method and the angle of the matching ground truth needle. The tip error is defined as the Euclidean distance between the tip of the needle estimated using our proposed method and the tip position in the matching ground truth needle. In fact, the failure rate is computed for the needle localization trials performed at shallow ($0^{\circ }$–$20^{\circ }$), moderate ($20^{\circ }$–$40^{\circ }$) and steep ($40^{\circ }$–$65^{\circ }$) needle insertion angles, as well as the entire range ($0^{\circ }$–$65^{\circ }$) of needle insertion angles considered in the current study. Furthermore, the mean ± standard deviation values of the angle, axis and tip errors are computed for the successful needle localization trials, i.e., the needle localization trials in which the needle axis error is less than 3 mm, that are performed at the different ranges of needle insertion angles described above.

In addition to our proposed method, we have implemented the two previous needle localization methods introduced by Hacihaliloglu et al. [12] and Greer et al. [21] and applied these two methods on the ultrasound images acquired for the needles. Moreover, we have computed the failure rate and the mean ± standard deviation values of the axis error, the angle error and the tip error for these two previous methods. Similar to our proposed method, the computation of the failure rate and the mean ± standard deviation values of the angle, axis and tip errors is performed for the shallow, moderate and steep needle insertion angles, as well as the entire range of needle insertion angles. Furthermore, the computation of the mean ± standard deviation values of the axis error, the angle error and the tip is carried out based on the successful needle localization trials in which the needle axis error is less than 3 mm. As described in the Introduction, the method by Greer et al. [21] was originally designed to estimate the 3D location of the needle by analyzing the power Doppler ultrasound images and the B-mode ultrasound images acquired for the excited needle using a tracked 2D ultrasound transducer. In the current study, the method by Greer et al. [21] has been employed to localize the needle in a pair of 2D power Doppler and 2D B-mode ultrasound images by eliminating the mapping of the needle candidate points to the 3D spatial coordinate system.

Our proposed needle localization method and the two previous needle localization methods in [12,21] are also evaluated in terms of their execution times. In particular, we have implemented these three methods using MATLAB (MathWorks Inc., Natick, MA, USA) and ran each method on a computer workstation with an Intel Xeon 2.6-GHz processor and 16 GB of memory. The mean ± standard deviation execution time of each one of the three needle localization methods is computed over all needle localization trials.

### 3.4. Analyzing the Effect of the Needle Insertion Depth on the Accuracy of Localizing the Needle

The set of 117 pairs of power Doppler and B-mode ultrasound images, which are described in Section 3.3, is employed to investigate the effect of varying the needle insertion depth on the needle localization accuracy obtained by our proposed method. As described previously, the insertion depths of the 18G needles included in the set of 117 pairs of power Doppler and B-mode images are approximately uniformly distributed between 40 and 80 mm. Therefore, we have computed the mean ± standard deviation values of the angle, axis and tip errors for the successful needle localization trials obtained by our proposed method when the needle insertion depth is within the ranges of 40–50 mm, 50–60 mm, 60–70 mm and 70–80 mm.

### 3.5. Analyzing the Effect of the Needle Size on the Accuracy of Localizing the Needle

To evaluate the effect of changing the needle size on the accuracy of our proposed method, we have acquired a set of 39 pairs of power Doppler and B-mode ultrasound images for 16G needles that are inserted in *ex vivo* bovine muscle tissue specimens. Similar to the ultrasound imaging producer described in Section 3.3, the acquisition of the 39 image pairs is performed such that the needle insertion angles are uniformly distributed between $0^{\circ }$ and $65^{\circ }$ and the needle insertion depths are between 40 and 80 mm. For each acquired image pair, our proposed method is employed to localize the needle. Furthermore, the localized needle in each image pair is compared with the matching ground truth needle that is obtained as described in Section 3.2. The failure rates are computed for the needle localizations conducted at shallow, moderate and steep needle insertion angles, as well as the entire range of needle insertion angles considered in the current study. In addition, the mean ± standard deviation values of the angle, axis and tip errors are calculated for the successful needle localizations that are performed at the four ranges of needle insertion angles described above.

## 4. Results

### 4.1. Results of the Performance Evaluations and Comparisons

Table 1 provides the needle localization results obtained by our proposed method for the 18G needles that were inserted in the three animal tissue types. The results show that our proposed method was able to localize the needles with a failure rate of 0.0% for the shallow, moderate and steep needle insertion angles. In fact, the values of the needle axis error did not exceed 1 mm for the three ranges of needle insertion angles described above.

In terms of the accuracy of localizing the needle, Table 1 shows that for the three animal tissue types, our proposed method was able to localize the needles inserted at shallow, moderate and steep insertion angles with mean angle errors between $0.2^{\circ }$ and $0.8^{\circ }$, mean axis errors between 0.2 and 0.6 mm and mean tip errors between 0.3 and 0.6 mm. When the entire range (0–$65^{\circ }$) of needle insertion angles is considered, our proposed method obtained mean angle errors between $0.4^{\circ }$ and $0.5^{\circ }$, mean axis errors between 0.3 and 0.4 mm and mean tip errors between 0.4 and 0.5 mm. These results indicate that the needle localization accuracy obtained by our proposed method degrades slightly when the insertion angle of the needle is increased. It is worth noting that for all needle insertion angles considered in this study, the values of the angle, axis and tip errors computed for our proposed method were obtained without excluding any needle localization trial since the failure rates are equal to 0.0% regardless of the needle insertion angle. These results demonstrate the capability of our proposed method to achieve reliable and accurate needle localization in curvilinear ultrasound images for an extended range of needle insertion angles.

The performance results obtained by the method in [12] are summarized in Table 2. The results show that for the shallow and moderate needle insertion angles, the method in [12] was able to localize the needles inserted in the three animal tissue types with failure rates of 0.0%. However, for the steep needle insertion angles, the failure rates of the method were between 40.0% and 33.3%. In fact, for the needles inserted in bovine muscle and porcine muscle at steep insertion angles, the method in [12] succeeded to localize all needles that have insertion angles smaller than $55^{\circ }$ and failed to localize all needles that have insertion angles greater than or equal to $55^{\circ }$. For the needles inserted in bovine liver at steep insertion angles, the method in [12] succeeded to localize all needles that have insertion angles smaller than $55^{\circ }$ and failed to localize all but one of the needles that have insertion angles greater than or equal to $55^{\circ }$. In terms of the needle localization accuracy, the method in [12] localized the needles inserted at shallow, moderate and steep insertion angles with mean angle errors between $0.9^{\circ }$ and $2.9^{\circ }$, mean axis errors between 1.1 and 2.4 mm and mean tip errors between 1.1 and 2.5 mm. When the entire range of needle insertion angles is considered, the method in [12] obtained mean angle errors between $1.8^{\circ }$ and $2.2^{\circ }$, mean axis errors between 1.5 and 1.7 mm and mean tip errors between 1.6 and 1.8 mm. It is worth noting that the values of the angle, axis and tip errors computed for the method in [12] at the shallow and moderate needle insertion angles were obtained based on all needle localization trials since the failure rates achieved at these two ranges of needle insertion angles were equal to 0.0%. However, for the steep needle insertion angles, the angle, axis and tip errors were calculated using the successful needle localization trials, which represent 60.0–66.7% of the needle localizations. Similarly, the angle, axis and tip errors reported for the entire range of needle insertion angles were computed based on the successful needle localization trials that represent 84.6–87.2% of the needle localizations. The results provided in Table 2 show that the needle localization reliability and accuracy obtained by the method in [12] were lower than our proposed method. Moreover, the results indicate that the performance of the method in [12] degraded drastically at steep needle insertion angles, particularly when the needle insertion angle exceeded $55^{\circ }$.

The results achieved by the method in [21] are presented in Table 3. Similar to the method in [12], the needle localization method in [21] was able to localize all needles inserted at shallow and moderate angles. However, for the steep needle insertion angles, the failure rates of the method in [21] were between 40.0% and 46.7%. In fact, for the needles inserted at steep angles in bovine muscle, the method in [21] succeeded to localize all but one of the needles that had insertion angles between $40^{\circ }$ and $55^{\circ }$ and failed to localize all needles with insertion angles between $55^{\circ }$ and $65^{\circ }$. For bovine liver and porcine muscle, the method in [21] succeeded to localize all needles with insertion angles between $40^{\circ }$ and $55^{\circ }$ and failed to localize all needles that had insertion angles between $55^{\circ }$ and $65^{\circ }$. In terms of the needle localization accuracy, the method in [21] localized the needles inserted at shallow, moderate and steep insertion angles with mean angle errors between $1.7^{\circ }$ and $2.9^{\circ }$, mean axis errors between 1.0 and 2.0 mm and mean tip errors between 1.1 and 2.1 mm. When the entire range of needle insertion angles is considered, the method in [21] obtained mean angle errors between $2.0^{\circ }$ and $2.3^{\circ }$, mean axis errors between 1.4 and 1.6 mm and mean tip errors between 1.5 and 1.6 mm. In fact, the values of the angle, axis and tip errors computed for the method in [21] at the shallow and moderate needle insertion angles were achieved using all needle localization trials since the failure rates at these two ranges of needle insertion angle were equal to 0.0%. For the steep needle insertion angles, the angle, axis and tip errors were obtained based on the successful needle localization trials, which represented between 53.3% and 60.0% of the needle localizations. In addition, the values of the angle, axis and tip errors reported for the entire range of needle insertion angles were achieved based on 82.1–84.6% of the needle localizations. Similar to the method in [12], the needle localization results obtained by the method in [21] were lower, in terms of both the reliability and accuracy, than the results achieved by the proposed method. Furthermore, the results reported for the method in [21] show that the performance of this method dropped at steep needle insertion angles, especially when the needle insertion angle was higher than $55^{\circ }$.

**Figure 6:**
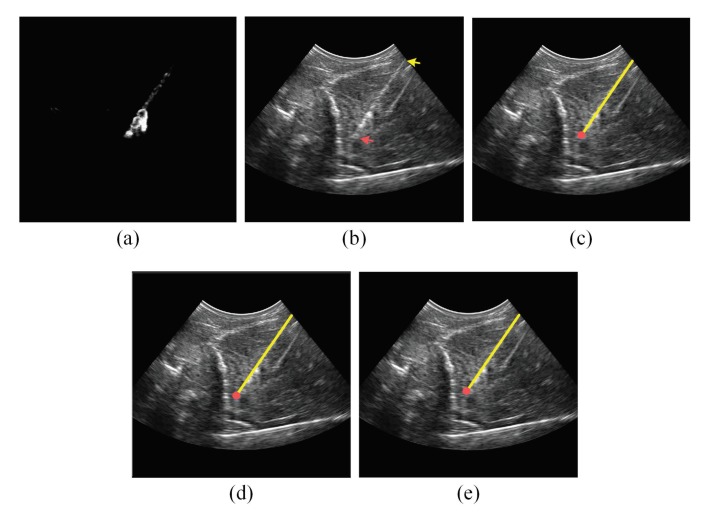
(**a**) Power Doppler ultrasound image and (**b**) B-mode ultrasound image acquired for an 18G needle inserted in bovine muscle at 54${}^{\circ }$. In the B-mode image, the yellow arrow points to the needle shaft and the red arrow points to the needle tip. (**c**) The needle localization result obtained by the proposed method. (**d**) The needle localization result obtained by the method in [12]. (**e**) The needle localization result obtained by the method in [21]. In (c–e), the localized needle axis and tip are shown as a yellow line and red circle, respectively, overlaid on the B-mode ultrasound image.

**Figure 7:**
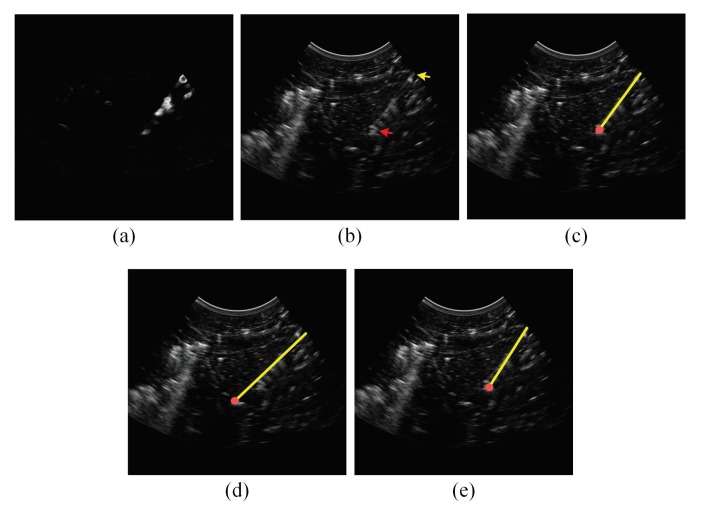
(**a**) Power Doppler ultrasound image and (**b**) B-mode ultrasound image acquired for an 18G needle inserted in bovine liver at 53${}^{\circ }$. In the B-mode image, the yellow arrow points to the needle shaft and the red arrow points to the needle tip. (**c**) The needle localization result obtained by the proposed method. (**d**) The needle localization result obtained by the method in [12]. (**e**) The needle localization result obtained by the method in [21]. In (c–e), the localized needle axis and tip are shown as a yellow line and a red circle, respectively, overlaid on the B-mode ultrasound image.

**Figure 8:**
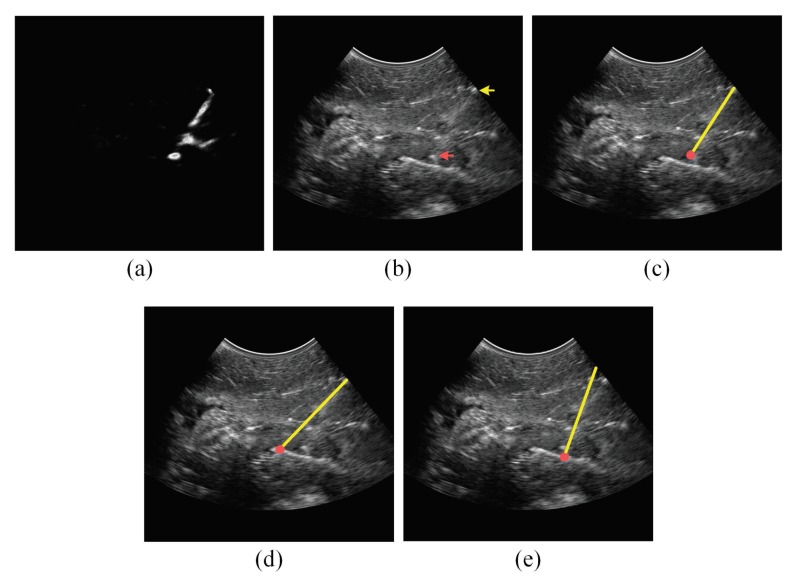
(**a**) Power Doppler ultrasound image and (**b**) B-mode ultrasound image acquired for an 18G needle inserted in porcine muscle at 59${}^{\circ }$. In the B-mode image, the yellow arrow points to the needle shaft and the red arrow points to the needle tip. (**c**) The needle localization result obtained by the proposed method. (**d**) The needle localization result obtained by the method in [12]. (**e**) The needle localization result obtained by the method in [21]. In (c–e), the localized needle axis and tip are shown as a yellow line and a red circle, respectively, overlaid on the B-mode ultrasound image.

**Figure 9:**
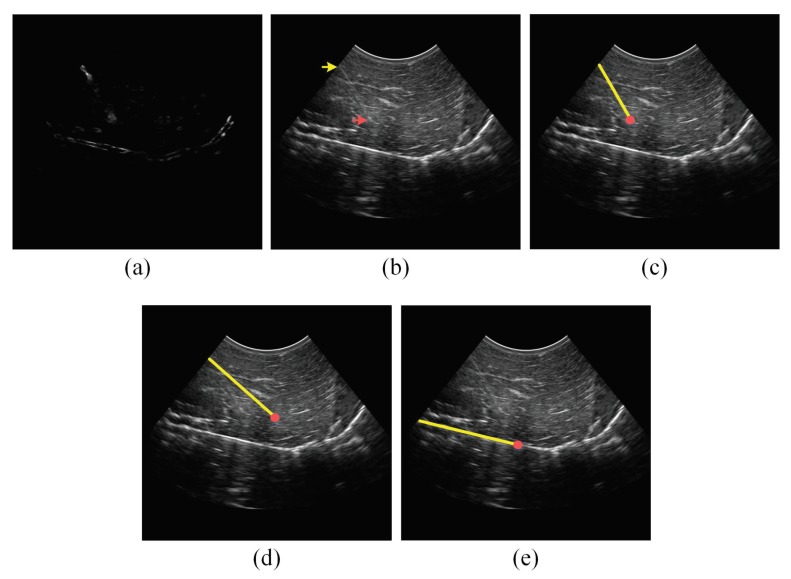
(**a**) Power Doppler ultrasound image and (**b**) B-mode ultrasound image acquired for an 18G needle inserted in bovine muscle at 60${}^{\circ }$. In the B-mode image, the yellow arrow points to the needle shaft and the red arrow points to the needle tip. (**c**) The needle localization result obtained by the proposed method. (**d**) The needle localization result obtained by the method in [12]. (**e**) The needle localization result obtained by the method in [21]. In (c–e), the localized needle axis and tip are shown as a yellow line and a red circle, respectively, overlaid on the B-mode ultrasound image.

In addition to the qualitative accuracy results provided in Table 1, Table 2 and Table 3, Figure 6, Figure 7, Figure 8 and Figure 9 show the needle localizations obtained by the proposed method and the previous methods in [12,21] for four 18G needles inserted in different tissue types at steep insertion angles. In fact, since the problem of localizing the needle at steep needle insertion angles is more challenging than localizing the needle at shallow and moderate insertion angles, Figure 6, Figure 7, Figure 8 and Figure 9 are focused on proving qualitative results for needles inserted at steep angles. In particular, the needles in Figure 6 and Figure 7 were inserted in bovine muscle tissue at an insertion angle of 54${}^{\circ }$ and bovine liver tissue at an insertion angle of 53${}^{\circ }$, respectively. The needles in Figure 8 and Figure 9 were inserted in porcine muscle tissue at an insertion angle of 59${}^{\circ }$ and bovine muscle tissue at an insertion angle of 60${}^{\circ }$, respectively. In each one of these four figures, Subfigure (a) is the power Doppler ultrasound image acquired for the inserted needle, (b) is the B-mode ultrasound image, (c) is the needle localization result obtained by the proposed method, (d) is the needle localization result achieved by the method in [12] and (e) is the needle localization result obtained by the method in [21]. As shown in the figures, the visibility of the needle at the insertion angles of 59${}^{\circ }$ and 60${}^{\circ }$ (Figure 8 and Figure 9) is lower than the visibility of the needle at the insertion angles of 53${}^{\circ }$ and 54${}^{\circ }$ (Figure 6 and Figure 7). For each one of the four figures, the needle axis and tip estimated by the proposed method were close to the location of the true needle, which is indicated by the arrows shown in the B-mode image in Subfigure (b). Moreover, the figures show that the needle localization results obtained by our proposed method outperformed the results achieved by the methods in [12,21].

In terms of the time needed to process the ultrasound image and localize the needle, the mean ± standard deviation execution time of our proposed method was 0.88±0.28 s. In comparison, the mean ± standard deviation execution times of the methods in [12,21] were 0.71±0.02 s and 0.39±0.14 s, respectively.

### 4.2. Results of Analyzing the Effect of the Needle Insertion Depth on the Accuracy of Localizing the Needle

As described in the previous subsection, our proposed method succeeded in localizing the needles in all 117 power Doppler and B-mode image pairs that were acquired for 18G needles inserted in the three animal tissue types. Hence, the entire set of 117 image pairs was employed to evaluate the effect of the needle insertion depth on the localization accuracy obtained by our proposed method.

Table 4 provides the mean ± standard deviation values of the angle, axis and tip errors obtained by our proposed method when the needle insertion depth was between 40 and 50 mm, 50 and 60 mm, 60 and 70 mm and 70 and 80 mm. In general, the results reported in Table 4 indicate that increasing the needle insertion depth leads to a slight reduction in the accuracy of localizing the needle. For the four ranges of needle insertion depths, the mean values of the angle error, axis error and tip error are between 0.3${}^{\circ }$ and 0.6${}^{\circ }$, 0.3 and 0.5 mm and 0.3 and 0.6 mm, respectively.

### 4.3. Results of Analyzing the Effect of the Needle Size on the Accuracy of Localizing the Needle

Table 5 provides the needle localization results achieved by our proposed method for the 39 pairs of power Doppler and B-mode ultrasound images acquired for the 16G needles that were inserted in bovine muscle tissue. Similar to the results obtained for the 18G needles, our proposed method succeeded in localizing the needles in all 39 image pairs with a failure rate of 0.0%. In fact, our proposed method achieved needle axis error values smaller than 1 mm for all 39 image pairs. Table 5 also shows that the needle localizations obtained by our proposed method at shallow, moderate and steep needle insertion angles had mean angle errors between 0.2${}^{\circ }$ and 0.7${}^{\circ }$, mean axis errors between 0.2 and 0.5 mm and mean tip errors between 0.3 and 0.6 mm. Furthermore, the mean values of the angle, axis and tip errors that were computed by considering the entire range of needle insertion angles were equal to 0.4${}^{\circ }$, 0.4 mm and 0.4 mm, respectively. Similar to the results reported for the 18G needles, Table 5 indicates that increasing the needle insertion angle leads to a slight decrease in the accuracy of our proposed method to localize the 16G needles. In general, the needle localization results reported in Table 5 for the 16G needles are close to the needle localization results provided in Table 1 that are obtained by our proposed method for the 18G needles.

## 5. Discussion

One important factor that affects the needle visibility in curvilinear ultrasound images is the needle insertion angle, where the visibility of the needle drops when the needle is inserted at steep angles [30]. In the current study, we have evaluated the capability of our proposed method and the two previous methods in [12,21] to localize the needles at shallow ($0^{\circ }$–$20^{\circ }$), moderate ($20^{\circ }$–$40^{\circ }$) and steep ($40^{\circ }$–$65^{\circ }$) needle insertion angles. In fact, the selection of these three ranges of needle insertion angles is based on the fact that the 4DC7-3/40 curvilinear ultrasound transducer, which is used to acquire the pairs of power Doppler and B-mode ultrasound images, has an aperture angle of $40^{\circ }$. Hence, for the shallow and moderate needle insertion angles, the ultrasound beams transmitted by the curvilinear ultrasound transducer can intercept perpendicularly with part of the needle shaft and hence generate strong spectral reflection at the needle. However, the opportunity to obtain ultrasound beams that intercept perpendicularly with the needle decreases at the range of steep needle insertion angles since the insertion angle of the needle is higher than the aperture angle of the transducer. Due to this limitation, the capability of localizing the needle at the range of steep needle insertion angles is usually reduced compared to the ranges of shallow and moderate needle insertion angles. In fact, the results provided in Table 1 show that our proposed method was able to localize the needles with high reliability and accuracy for the entire range of insertion angles considered in this study, but the performance of the proposed method was slightly degraded when steep needle insertion angles were employed. On the other hand, the results in Table 2 and Table 3 that are computed for the methods in [12,21] are lower than the results obtained by our proposed method. Table 2 and Table 3 also indicate that the use of steep needle insertion angles led to drastic degradation in the capabilities of these two previous methods to localize the needles. Furthermore, the performance results reported for our proposed needle localization method and the two previous methods in [12,21] were limited to the needle insertion angles between $0^{\circ }$ and $65^{\circ }$. In fact, our analysis indicated that none of these three needle localization methods were able to detect the needle when the needle insertion angle was increased to values higher than $65^{\circ }$. This can be attributed to the fact that increasing the needle insertion angle to values higher than $65^{\circ }$, which corresponds to $25^{\circ }$ greater than the aperture angle of the 4DC7-3/40 curvilinear ultrasound transducer, led to high degradation in the visibility of the needle in the B-mode image and a drastic drop in the capability of the power Doppler image to detect the needle-induced Doppler responses.

Consequently, an important element that affects the capability of our proposed method to localize the needle in curvilinear ultrasound images acquired by ultrasound imaging systems that are different from the ultrasound system employed in the current study is the aperture angle of the transducer. In particular, the use of ultrasound transducers with aperture angles greater than 40${}^{\circ }$ is expected to increase the range of needle insertion angles in which the needle can be localized with high reliability and accuracy, and vice versa. Another important element that might affect the performance of the proposed method is the capability of the imaging system to detect the Doppler responses induced by the excited needle. In fact, the capability of the ultrasound imaging system to detect the needle-induced vibrations is mainly affected by the piezoelectric buzzer employed to excite the needle and the Doppler imaging configurations, particularly the PRF and the wall motion filter, of the ultrasound system.

Another crucial factor that affects the visibility of the needle in curvilinear ultrasound images is the needle insertion depth. In fact, the lateral resolution of curvilinear ultrasound images degrades as a function of depth, which leads to blurring and deformation artifacts [31]. Furthermore, the amplitude of the Doppler responses generated by the excited needle might be reduced when the needle is inserted at high depths due to the damping of the needle vibrations by the surrounding tissue. Despite the depth-dependent degradation in the lateral resolution, many previous studies, such as [12,14], that proposed needle detection methods based on B-mode ultrasound image analysis showed that the needle can be localized in curvilinear ultrasound images with submillimeter accuracies. In fact, the method presented in the current study was also able to obtain accurate needle localization with submillimeter accuracy by combining power Doppler and B-mode ultrasound image analyses. The results reported in Table 4 indicate that for the range of needle insertion depths considered in the current study (40–80 mm), increasing the insertion depth of the needle reduced the needle localization accuracy slightly. This reduction in the needle localization accuracy might be attributed to the depth-dependent degradation in the lateral resolution and the potential depth-dependent decrease in the Doppler responses induced by the excited needle. In fact, one of our future directions is to study the performance of our proposed method when the needle is inserted at depths higher than 80 mm and expand our proposed method to achieve reliable and accurate needle localization at high needle insertion depths.

The visibility of the needle in ultrasound images might be also affected by the size of the needle. In fact, the study by Schafhalter-Zoppoth et al. [30] indicated that the needle visibility in ultrasound images is related to the needle insertion angle and the needle size. In the current study, we have employed the proposed method to localize needles with two different sizes, namely the 18G and 16G needles, at various needle insertion angles. The results reported in Table 1 and Table 5 show that our proposed method achieved reliable and accurate localization of the 18G and 16G needles at shallow, moderate and steep needle insertion angles. In the future, we are planning to investigate the capability of our proposed method to localize additional needle types that have different sizes. In particular, we are interested in evaluating the reliability and accuracy of our proposed method to localize fine needles, such as the 22G needles, that have low visibility in ultrasound images.

The promising results reported in the current study suggest the potential of applying our proposed method to achieve automatic, reliable and accurate needle localization during ultrasound-guided needle interventions. In fact, a major advantage of our proposed method is the low cost and the limited equipment that are needed to implement the method. Compared to conventional ultrasound-based needle localization methods, the additional equipment that is required to apply our proposed method includes the piezoelectric buzzer and function generator that are employed to excite the needle and the power Doppler ultrasound imaging feature that exists in most modern ultrasound imaging systems. However, one limitation that might restrict the application of the proposed method in clinical needle interventions is the possible influence of the Doppler signals created by blood flow and organs’ movement on the capability of identifying the Doppler responses generated by the excited needle. Furthermore, the needle-induced vibrations can be affected by the heterogeneity of the surrounding tissues that might have different mechanical properties. To the best of our knowledge, the previous methods that employed Doppler ultrasound imaging to localize excited needles did not include *in vivo* studies to investigate the limitations of the Doppler-based needle localization approach in realistic living conditions. Hence, in the future, we are planning to extend the evaluations of our proposed method to include *in vivo* studies and expand the method to enable reliable and accurate needle localization in realistic living conditions. For example, the needle can be excited at high-frequency bands to generate unique needle-induced Doppler responses that can be differentiated from the Doppler singles created by blood flow and organs’ movement.

Another limitation that might restrict the application of our proposed method in clinical settings is the execution time that has a mean value of 0.88 s. To overcome this limitation, we are planning to implement our proposed method using graphics processing unit (GPU) technology to reduce its running time and support needle localization at interactive rates.

## 6. Conclusions

This study aims to introduce a new method to enable automatic, reliable and accurate needle localization in 2D curvilinear ultrasound images by combining power Doppler and B-mode ultrasound image analyses. In particular, the needle is excited using acoustic waves generated by a piezoelectric buzzer mounted near the needle base to induce power Doppler responses around the needle. The excited needle is imaged using a curvilinear 2D ultrasound transducer to acquire a power Doppler image and a B-mode image. The power Doppler image, which detects the needle-induced power Doppler responses, is analyzed to obtain an initial estimation of the needle axis and determine ROIs, called the power Doppler-based ROIs, in the image that are expected to include the needle. The accuracy of the initially estimated needle axis is improved by analyzing the B-mode image. In particular, the B-mode image is processed using a Gabor filter to improve the visibility of the linear structures in the image that match the orientation of the initially estimated needle axis and the known scale of the needle. The filtering process of the B-mode image using the Gabor filter is masked using the power Doppler-based ROIs. The filtered B-mode image is analyzed using Radon transform to obtain accurate localization of the needle axis. The position of the needle tip is determined by analyzing the intensity variations of the power Doppler image and the B-mode image around the localized needle axis. In particular, approximate localization of the needle tip is obtained by analyzing the region that surrounds the needle axis in the power Doppler image to find the termination point that separates the strong power Doppler responses induced by the needle from the weak power Doppler responses that are not related to the needle. Moreover, the accuracy of localizing the needle tip is improved by analyzing the region that surrounds the needle axis in the B-mode image to find the point that separates the bright pixels of the needle from the pixels of the background tissue. The performance of the proposed method has been evaluated by localizing the axes and tips of different needles that are inserted in three *ex vivo* animal tissue types at shallow, moderate and steep needle insertion angles. The results reported in the current study demonstrate the capability of our proposed method to achieve automatic, accurate and reliable needle localization in curvilinear ultrasound images.

## Figures and Tables

**Table 1:** The failure rate, axis error, angle error and tip error results obtained by the proposed method for 18G needles inserted in three types of animal tissues as a function of the needle insertion angle.

Range of Needle Insertion Angles	Tissue Type	Failure Rate	Angel Error (${}^{\circ }$)	Axis Error (mm)	Tip Error (mm)
Shallow angles ($0^{\circ }$–$20^{\circ }$)	Bovine muscle	0.0%	0.2 ± 0.1	0.3 ± 0.1	0.3 ± 0.1
	Bovine liver	0.0%	0.2 ± 0.1	0.2 ± 0.1	0.3 ± 0.1
	Porcine muscle	0.0%	0.2 ± 0.1	0.2 ± 0.1	0.3 ± 0.1
Moderate angles ($20^{\circ }$–$40^{\circ }$)	Bovine muscle	0.0%	0.4 ± 0.2	0.4 ± 0.1	0.5 ± 0.1
	Bovine liver	0.0%	0.3 ± 0.2	0.3 ± 0.1	0.4 ± 0.1
	Porcine muscle	0.0%	0.3 ± 0.2	0.4 ± 0.1	0.4 ± 0.1
Steep angles ($40^{\circ }$–$65^{\circ }$)	Bovine muscle	0.0%	0.8 ± 0.2	0.5 ± 0.1	0.6 ± 0.1
	Bovine liver	0.0%	0.7 ± 0.2	0.5 ± 0.1	0.5 ± 0.1
	Porcine muscle	0.0%	0.8 ± 0.2	0.6 ± 0.1	0.5 ± 0.1
All angles ($0^{\circ }$–$65^{\circ }$)	Bovine muscle	0.0%	0.5 ± 0.3	0.4 ± 0.1	0.5 ± 0.1
	Bovine liver	0.0%	0.4 ± 0.3	0.3 ± 0.2	0.4 ± 0.1
	Porcine muscle	0.0%	0.5 ± 0.3	0.4 ± 0.2	0.4 ± 0.1

**Table 2:** The failure rate, axis error, angle error and tip error results obtained by the previous method introduced in [12] for 18G needles inserted in three types of animal tissues as a function of the needle insertion angle.

Range of Needle Insertion Angles	Tissue Type	Failure Rate	Angel Error (${}^{\circ }$)	Axis Error (mm)	Tip Error (mm)
Shallow angles ($0^{\circ }$–$20^{\circ }$)	Bovine muscle	0.0%	0.9 ± 0.6	1.1 ± 0.3	1.1 ± 0.4
	Bovine liver	0.0%	1.3 ± 0.5	1.2 ± 0.5	1.3 ± 0.5
	Porcine muscle	0.0%	1.1 ± 0.4	1.1 ± 0.3	1.2 ± 0.3
Moderate angles ($20^{\circ }$–$40^{\circ }$)	Bovine muscle	0.0%	1.9 ± 0.4	1.7 ± 0.2	1.6 ± 0.2
	Bovine liver	0.0%	2.5 ± 1.2	1.8 ± 0.5	1.8 ± 0.7
	Porcine muscle	0.0%	2.2 ± 1.2	1.5 ± 0.4	1.6 ± 0.6
Steep angles ($40^{\circ }$–$65^{\circ }$)	Bovine muscle	40.0%	2.9 ± 0.6	2.3 ± 0.3	2.2 ± 0.4
	Bovine liver	33.3%	2.9 ± 1.2	2.4 ± 0.4	2.5 ± 0.5
	Porcine muscle	40.0%	2.7 ± 1.1	2.0 ± 0.5	2.1 ± 0.5
All angles ($0^{\circ }$–$65^{\circ }$)	Bovine muscle	15.4%	1.8 ± 1.0	1.7 ± 0.5	1.6 ± 0.5
	Bovine liver	12.8%	2.2 ± 1.2	1.7 ± 0.7	1.8 ± 0.7
	Porcine muscle	15.4%	1.9 ± 1.2	1.5 ± 0.5	1.6 ± 0.6

**Table 3:** The failure rate, axis error, angle error and tip error results obtained by the previous method introduced in [21] for 18G needles inserted in three types of animal tissues as a function of the needle insertion angle.

Range of Needle Insertion Angles	Tissue Type	Failure Rate	Angel Error (${}^{\circ }$)	Axis Error (mm)	Tip Error (mm)
Shallow angles ($0^{\circ }$–$20^{\circ }$)	Bovine muscle	0.0%	1.7 ± 0.9	1.2 ± 0.6	1.2 ± 0.7
	Bovine liver	0.0%	1.8 ± 0.8	1.0 ± 0.4	1.1 ± 0.4
	Porcine muscle	0.0%	1.8 ± 0.3	1.1 ± 0.5	1.1 ± 0.5
Moderate angles ($20^{\circ }$–$40^{\circ }$)	Bovine muscle	0.0%	2.0 ± 1.0	1.6 ± 0.7	1.6 ± 0.8
	Bovine liver	0.0%	2.2 ± 1.1	1.5 ± 0.7	1.6 ± 0.9
	Porcine muscle	0.0%	2.3 ± 0.7	1.7 ± 0.6	1.8 ± 0.8
Steep angles ($40^{\circ }$–$65^{\circ }$)	Bovine muscle	46.7%	2.6 ± 1.2	1.9 ± 0.5	2.1 ± 0.3
	Bovine liver	40.0%	2.7 ± 1.0	1.9 ± 0.7	2.0 ± 0.5
	Porcine muscle	40.0%	2.9 ± 1.5	2.0 ± 0.4	2.0 ± 0.5
All angles ($0^{\circ }$–$65^{\circ }$)	Bovine muscle	17.9%	2.0 ± 1.1	1.5 ± 0.7	1.6 ± 0.7
	Bovine liver	15.4%	2.2 ± 1.0	1.4 ± 0.7	1.5 ± 0.7
	Porcine muscle	15.4%	2.3 ± 1.0	1.6 ± 0.6	1.6 ± 0.7

**Table 4:** The axis error, angle error and tip error results obtained by the proposed method for 18G needles inserted in three types of animal tissues as a function of the needle insertion depth.

Range of Needle Insertion Angles	Tissue Type	Angel Error (${}^{\circ }$)	Axis Error (mm)	Tip Error (mm)
(40mm–50mm)	Bovine muscle	0.4 ± 0.2	0.3 ± 0.2	0.3 ± 0.1
	Bovine liver	0.3 ± 0.2	0.3 ± 0.1	0.3 ± 0.1
	Porcine muscle	0.3 ± 0.2	0.3 ± 0.2	0.4 ± 0.1
(50mm–60mm)	Bovine muscle	0.5 ± 0.3	0.4 ± 0.1	0.5 ± 0.2
	Bovine liver	0.3 ± 0.2	0.3 ± 0.1	0.4 ± 0.1
	Porcine muscle	0.4 ± 0.2	0.4 ± 0.1	0.4 ± 0.1
(60mm–70mm)	Bovine muscle	0.5 ± 0.3	0.4 ± 0.2	0.5 ± 0.2
	Bovine liver	0.5 ± 0.2	0.4 ± 0.2	0.4 ± 0.1
	Porcine muscle	0.5 ± 0.2	0.4 ± 0.1	0.4 ± 0.1
(70mm–80mm)	Bovine muscle	0.6 ± 0.3	0.5 ± 0.2	0.6 ± 0.2
	Bovine liver	0.5 ± 0.3	0.4 ± 0.2	0.5 ± 0.2
	Porcine muscle	0.6 ± 0.2	0.5 ± 0.1	0.5 ± 0.1

**Table 5:** The failure rate, axis error, angle error and tip error results obtained by the proposed method for 16G needles inserted in bovine muscle tissue as a function of the needle insertion angle.

Range of Needle Insertion Angles	Failure Rate	Angel Error (${}^{\circ }$)	Axis Error (mm)	Tip Error (mm)
Shallow angles ($0^{\circ }$–$20^{\circ }$)	0.0%	0.2 ± 0.1	0.2 ± 0.1	0.3 ± 0.1
Moderate angles ($20^{\circ }$–$40^{\circ }$)	0.0%	0.3 ± 0.1	0.4 ± 0.1	0.4 ± 0.1
Steep angles ($40^{\circ }$–$65^{\circ }$)	0.0%	0.7 ± 0.2	0.5 ± 0.1	0.6 ± 0.1
All angles ($0^{\circ }$–$65^{\circ }$)	0.0%	0.4 ± 0.3	0.4 ± 0.2	0.4 ± 0.2
